# The FDA’s Experience with Emerging Genomics Technologies—Past, Present, and Future

**DOI:** 10.1208/s12248-016-9917-y

**Published:** 2016-04-26

**Authors:** Joshua Xu, Shraddha Thakkar, Binsheng Gong, Weida Tong

**Affiliations:** 1 Division of Bioinformatics and Biostatistics, National Center for Toxicological Research, U.S. Food and Drug Administration, Jefferson, Arkansas, USA

**Keywords:** big data, genomics, next-generation sequencing, reproducibility, RNA-seq

## Abstract

The rapid advancement of emerging genomics technologies and their application for assessing safety and efficacy of FDA-regulated products require a high standard of reliability and robustness supporting regulatory decision-making in the FDA. To facilitate the regulatory application, the FDA implemented a novel data submission program, Voluntary Genomics Data Submission (VGDS), and also to engage the stakeholders. As part of the endeavor, for the past 10 years, the FDA has led an international consortium of regulatory agencies, academia, pharmaceutical companies, and genomics platform providers, which was named MicroArray Quality Control Consortium (MAQC), to address issues such as reproducibility, precision, specificity/sensitivity, and data interpretation. Three projects have been completed so far assessing these genomics technologies: gene expression microarrays, whole genome genotyping arrays, and whole transcriptome sequencing (i.e., RNA-seq). The resultant studies provide the basic parameters for fit-for-purpose application of these new data streams in regulatory environments, and the solutions have been made available to the public through peer-reviewed publications. The latest MAQC project is also called the SEquencing Quality Control (SEQC) project focused on next-generation sequencing. Using reference samples with built-in controls, SEQC studies have demonstrated that relative gene expression can be measured accurately and reliably across laboratories and RNA-seq platforms. Besides prediction performance comparable to microarrays in clinical settings and safety assessments, RNA-seq is shown to have better sensitivity for low expression and reveal novel transcriptomic features. Future effort of MAQC will be focused on quality control of whole genome sequencing and targeted sequencing.

## INTRODUCTION

A decade ago, microarrays were the mainstream genomics technology used by the biomedical and pharmaceutical research communities. Using this technique, a large expanse of microarray data has been generated to support the drug development process. For example, genomics data has been used to evaluate drug safety and efficacy in support of both investigational new drug applications (IND) and new drug applications (NDA). To facilitate the submission of genomics data, the FDA created a novel data submission program known as Voluntary Genomics Data Submission (VGDS) and later extended it to Voluntary eXploratory Data Submission (VXDS) so that other omics data could be included. The idea behind this novel submission program was to facilitate FDA’s communication with the sponsor and to identify the best ways to apply omics data in regulatory application. The results of these efforts have helped to develop the Guidance for Industry on Pharmacogenomics (PGx) Data Submission (1). The VXDS program encourages the sponsor to interact with the FDA through submission of PGx data on a voluntary basis. In addition to that, it provides a forum for scientific discussions with the FDA outside of the regulatory review process. This whole process has helped to establish a regulatory environment within the FDA for receiving, analyzing, and interpreting the PGx data.

In order to achieve the goals of the VXDS process, the FDA created a *data repository* to keep track of all the data submitted by the sponsors (2). The submitted information was important to shape future regulatory policies regarding PGx data submission and review. In an effort to create new standards for receiving PGx data, the FDA sought to reproduce the analysis results and conclusions provided by the sponsor. In addition to that, alternative analysis and biological interpretation were also conducted and compared with the sponsor’s analysis. These efforts established FDA’s view for analysis and interpretation of PGx information.

During these efforts, it was identified that even the slightest change in the statistical methods could lead to substantial differences between the results from the sponsor and those from the agency (1, 2). Differences in the statistical analysis results led to discrepancies in biological interpretation. The high variations in analysis results were not just related to the microarray technology, but were also observed in most of the high-throughput screening technologies, including those utilized in proteomics and metabolomics.

Whenever a new technology is introduced to assist in the process of drug development, the biomedical and pharmaceutical research community tries to evaluate its potentials in understanding the underlying mechanisms of drug efficacy and toxicity. These evaluation efforts enhance the understanding of the utility of the technologies, and the research community learns their appropriate fit-for-purpose applications. However, it may take 15–20 years for an innovative technology to be translated to fit-for-purpose applications in a regulatory setting (3). It is thus of FDA’s interest to be involved in the evaluation efforts in order to expedite such translation. Through the efforts reviewed here, the FDA has demonstrated its commitment to expedite the process of incorporating the application of innovative technologies. These efforts were carried out in collaboration with the research community and stakeholders, with an emphasis on promoting the optimization, reproducibility, and standardization of the analysis protocol, data interpretation, and data sharing.

## MAQC CONSORTIUM

The MicroArray Quality Control (MAQC) consortium is a community-wide effort led by the FDA to address the above mentioned reproducibility concerns about the genomics technologies. It was started about 10 years ago, involving most FDA centers along with the international research community and industry. Its objective was to analyze the technical performance and utility of emerging molecular technologies (e.g., microarrays, next-generation sequencing) for clinical application and safety assessment. Throughout MAQC efforts, there was a consistent emphasis on transparency. The results and conclusions were published in peer-reviewed journals. The data generated during these efforts has been made freely available to the public. Additionally, some biological samples from which the data were generated are also available from commercial vendors. The consortium started in 2005 and by the end of 2014, three projects were completed. During the course of these projects, three different genomics technologies were evaluated. Under the project MAQC 1 and 2, microarrays were evaluated. Various issues related to the genome-wide genotyping arrays were evaluated in the MAQC 2 project (4–15). The third MAQC project, also known as SEquencing Quality Control (SEQC), evaluated the RNA-seq technology. All three projects evaluated the fit-for-purpose application for clinical and regulatory aspects of those genomics technologies. The entire project published a total of 28 peer-reviewed articles (http://www.fda.gov/ScienceResearch/BioinformaticsTools/MicroarrayQualityControlProject/), and 11 of them were published in *Nature Biotechnology* (4, 16-24). The paper published from MAQC 1 project supported the FDA in the development of “Guidance for Industry: Pharmacogenomics Data Submission – Companion Guidance.”

The MAQC 1 project demonstrated inter- and intraplatform reproducibility of gene expression measurements by microarrays. The comprehensive study design was centered on cross-site cross-platform performance evaluation through the titration of two reference RNA samples. DNA microarray results were compared with the quantitative PCR platforms for gene expression, and high correlation was observed between them. Additionally, external RNA controls for the assessment of microarray performance were also evaluated, along with various microarray data normalization techniques. Importantly, MAQC 1 studies demonstrated that the combination of fold-change ranking and a non-stringent *P* value cutoff led to increased consistency in differential gene expression analysis and downstream biological interpretation. The reference RNA samples chosen by the consortium have since become standard material widely adopted by the research community and the biotechnology industry for laboratory proficiency testing and development of new genomics technologies. As a natural progression, the MAQC 2 project studied the development and reliability of microarray-based predictive models for a variety of preclinical and clinical endpoints. Over 30,000 models were developed by 36 data analysis teams using numerous model building methods. Performance evaluation through a strictly blind external validation process demonstrated the utility of well-implemented internal cross validation in gauging the model prediction performance. This carefully designed and executed consortium effort with six large clinical and preclinical microarray datasets demonstrated that reliable predictive models can be developed when including sound and unbiased cross-validation techniques in the process. We expect the conclusions from the MAQC 2 project to be applicable to models based on gene expression data from other high-throughput technologies besides microarrays.

## CHALLENGES FOR NGS

The next-generation sequencing (NGS) technologies were first introduced to the market in 2005 and have since seen tremendous growth in both technology advancements and research adoption. NGS has a wide spectrum of application in biomedical research including but is not limited to genome and exome sequencing, whole transcriptome sequencing (i.e., RNA-seq), microRNA sequencing, and metagenomics. Some common challenges related to NGS include data storage, transfer, sharing, analysis, and visualization due to the sheer size of NGS datasets, which are referred to as big data challenges. As a tool, the specific application of NGS mainly defines the challenges and issues associated with this technique. Our literature survey on the use of NGS as a tool found that about 50% of the applications are mainly related to the use of NGS to understand genetic variations and their effect on disease and drug response. About a quarter of the applications are related to RNA-seq while the rest of them are split into various areas including microRNA sequencing and metagenomics. Challenges and issues associated with human genome sequencing differs greatly from these associated with microRNA sequencing because the size of the molecular object under investigation varies greatly, i.e., the human genome has 3.2 billion base pairs in contrast to the microRNA size of only 18–25 nucleotides.

## THIRD PHASE OF MAQC PROJECT

Due to rapid advances in NGS technologies, the third phase of the MAQC Project was initiated while the second phase was still under the way. As mentioned above, this phase is also known as the SEQC project with its focus on RNA-seq. Over 180 participants from 73 different organizations across 12 different countries participated in the SEQC project. The project generated over 10 TB of data with over 100 billion reads. On submission of this dataset to the Gene Expression Omnibus (GEO) repository in June 2014, it represented around 6% of the total RNA-seq data in the repository at that time. This rich data provides ample opportunities for RNA-seq data analysis method development. Under this project, four different datasets were generated. The first dataset was generated from six reference samples. These reference samples were sequenced by various laboratories using different RNA-seq platforms such as Illumina HiSeq, Life Technologies SOLiD, and Roche 454. The second dataset was composed of sequencing data for about 500 neuroblastoma samples from pediatric patients. The third dataset was from 100 rat liver samples. The last dataset was a survey of rat transcriptomes using 11 different organs across 4 different developmental stages for both male and female rat. The SEQC project evaluated technical performance, quality control, and cross lab and cross platform reproducibility of RNA-seq. RNA-seq data was also compared with data generated from the same samples by mature microarray technologies. In addition to that, evaluations were made on the use of RNA-seq for clinical applications and safety assessments. The observations from these efforts were published in 10 manuscripts (3, 22-30). Here we present five major findings:

1. Relative measurement is more consistent than absolute measurement.
  We generated large datasets for six reference samples. The samples were sequenced in 11 different laboratories using various platforms (i.e., HiSeq, SOLiD, and 454) (27) with multiple library preparation replicates for each sample at each laboratory. This study design offered us an opportunity to evaluate cross lab and cross platform consistency using the same sample. It allowed us to study both intra-laboratory and cross laboratory variability. Ideally, no gene would be differentially expressed for the same sample when it is sequenced with the same platform in different labs. We observed that as many as 10,000 genes could pass the statistical test to be considered as differentially expressed. In contrary, when differentially expressed genes (DEGs) from any pair of samples were compared across laboratories and platforms, the results were quite consistent (22). Thus, the analyses demonstrated that relative measurement is much more consistent and reproducible than the absolute measurement.
2. RNA-seq vs microarrays.
  Among its broad application, RNA-seq has two major applications, first is to determine DEGs by comparing different conditions, e.g., treatment or disease status. The second use is to develop gene expression-based predictive models. However, microarrays have been used for a long time to perform similar tasks. Bioinformatics methods for analyzing and interpreting the results from microarray data have been assessed and established through the first two MAQC projects. In comparison, RNA-seq is a relatively new technology and analysis methods are continuously being developed. Thus, there is a great interest in the community to compare microarrays and RNA-seq to identify the benefits of using RNA-seq over microarrays. To address the comparison, the SEQC project implemented several studies to comprehensively assess the difference and similarity between these two technologies. In one of them, rat livers treated with 15 chemicals and matched controls were profiled with both technologies and the DEGs detected for each chemical were compared between the two technologies. Of note, these chemicals yielded a wide range of treatment effect with a 10-fold difference between the smallest and largest number of DEGs detected. With this design, we could evaluate the concordance in DEG analysis between RNA-seq and microarrays in various levels of treatment effect. We found that the concordance in DEGs between microarray and RNA-seq was positively correlated with the strength of treatment effect. Further analyses indicated that the discordance was mostly due to the difference between two platforms in quantifying the lowly expressed genes. Specifically, for highly expressed genes, we were able to achieve a concordance of about 75% while the concordance was only 35% for lowly expressed genes. Thus, the major difference between microarrays and RNA-seq lies in their accuracy of measuring lowly expressed genes. Further comparison with quantitative PCR indicated that RNA-seq would likely perform better than microarrays for lowly expressed genes (24).
3. RNA-seq and Gene Discovery.
  An important potential of RNA-seq is its ability to discover novel, unannotated exon-exon junctions, which is affected by read depth. On increasing the number of reads from 10 million to 10 billion, both known genes and novel junctions were continually detected. Importantly, using quantitative PCR to validate some selected novel junctions, we determined that over 80% of them can be verified but their biological functions are unknown (14). This observation opens the door for the research community to peruse the area of increased read depth analysis and identify new transcripts and evaluate the contribution of such new transcripts or genes to understand the underlying biological mechanisms related to disease and toxicity.
4. Pipeline for RNA-seq.
  One of the most asked questions in the research community is which pipeline(s) is to be used for RNA-seq data analysis. To address the question in the context of big data, we evaluated 12 different pipelines in this project. For each pipeline, there are different parameter settings that lead to 278 major permutations covering the common gene modes, various quantification, and normalization methods. The comprehensive assessment was extremely costly in terms of computational time. We identified DEGs and compared the results with quantitative PCR and also evaluated the performance of downstream prediction models. We developed a composite metric including accuracy, precision, sensitivity in detecting lowly expressed genes, specificity in detecting DEGs, and prediction performance to derive the best practice for choosing RNA-seq data analysis pipelines. We observed that the pipeline giving the better estimation of the gene expression likely also gave better performance in predictive modeling. Multiple pipeline components jointly and significantly impacted the quantification of gene expression and downstream prediction performance. The manuscript is currently under review at *Nature Methods*.
5. Legacy microarray data in the RNA-seq era.
  Microarrays have been widely used in biomedical research and drug development since 1995. Major pharmaceutical companies usually generate thousands of microarrays per year. In this analysis, we tried to address whether RNA-seq-based gene signatures can be applied to microarray data to leverage the investment previously made. We tested three different classifier methods with three gene mapping categories to identify the transferability of microarray information to the RNA-seq data and vice versa. RNA-seq and microarrays were comparable for predictive models. Importantly, signature genes were reciprocally transferable between these two technology platforms. Microarray models can accurately predict RNA-seq-profiled samples. However, RNA-seq was less accurate in predicting microarray-profiled samples, and the performance was affected by modeling algorithms and the gene mapping complexity (26).

## PERSPECTIVES AND FUTURE DIRECTION

NGS technologies have emerged as an important tool for many regulatory activities. Various FDA centers have encountered NGS data in regulatory science research and/or regulatory applications. These include but are not limited to (i) FDA oversight of NGS-based assays for diagnosis and prognosis, (ii) applying NGS in food pathogen identification and outbreak detection, (iii) reviewing NGS data for drug efficacy and safety for both clinical and preclinical assessments, and (iv) NGS as an improved tool for studying immunogenicity of vaccines. More specifically for biological products, NGS data can be utilized in various ways to support their development with one current major use being the identification of microbial contaminations (31).

Building upon the success of the previous MAQC projects, which were fundamental for the development of FDA companion guidance to industry on pharmacogenomics data submission, we are in the process of developing a follow-up project, named SEquencing Quality Control Phase 2 (SEQC2). SEQC2 aims to develop quality control metrics and benchmark bioinformatics approaches for the analysis of the whole genome sequencing and targeted gene sequencing data to achieve best practices, to develop standard analysis protocols, and to apply these newer methods in regulatory settings. The ultimate goal of SEQC2 is the development of standards for using NGS data that will provide the FDA with objective criteria and metrics for data quality assessment that can be applied in regulatory settings and to provide information for precision medicine.

In summary, the primary aim of these FDA-led efforts for emerging genomics technologies is to engage the stakeholders and research community for consensus building with respect to the reliable use of genomics data with objective criteria and assessment metrics for data quality and reliability, which can be employed in the FDA for their fit-for-purpose application.
